# Is employment anxiety among vocal music students associated with AI replacement concerns? The roles of AI anxiety and vocal-performance replacement perception

**DOI:** 10.3389/fpsyg.2026.1923159

**Published:** 2026-08-14

**Authors:** Kehang Li, Yuheng Zhang, Wen Ji

**Affiliations:** College of Arts, Xiamen University, Xiamen, Fujian, China

**Keywords:** artificial intelligence anxiety, career direction clarity, employment anxiety, music education, vocal music students, vocal-performance replacement perception

## Abstract

Artificial intelligence (AI) is reshaping students' expectations about learning, employability, and professional identity, but AI-related psychological responses may differ across disciplines. This cross-sectional study examined how broad AI anxiety and a vocal-performance-specific replacement appraisal were associated with employment anxiety among 392 vocal music students and early-career graduates in China. AI anxiety was assessed with a 14-item subset derived from the Chinese Artificial Intelligence Anxiety Scale; vocal-performance replacement perception was assessed with a four-item context-adapted item set informed by the STARA awareness framework. AI anxiety was strongly associated with employment anxiety (*r* = 0.678, *p* < 0.001), whereas vocal-performance replacement perception showed a weaker zero-order association (*r* = 0.277, *p* < 0.001). Williams's test confirmed that the two dependent correlations differed, *t*_(389)_ = 10.23, *p* < 0.001. In a hierarchical regression controlling for education stage/status, school type, and AI-use experience, AI anxiety had a positive coefficient (β = 0.589, *p* < 0.001), while vocal-performance replacement perception did not explain additional variance (β = 0.010, *p* = 0.824). Perceived career-direction clarity had a negative coefficient (β = –0.174, *p* < 0.001). Sensitivity analyses that removed the AI-anxiety work-replacement dimension or only its explicit replacement items yielded the same pattern. Within the set of AI-related variables examined, employment anxiety in this sample was more closely associated with broad AI anxiety than with an explicit belief that AI will replace vocal performance. The findings do not imply that replacement concerns are irrelevant or establish causal direction.

## 1 Introduction

Artificial intelligence has become a central force in contemporary higher education and in the reorganization of future work. Students increasingly encounter AI as both a learning tool and a signal that professional knowledge, skill acquisition, and career entry may be changing. AI anxiety offers one way to understand students' psychological responses to technologies that appear powerful, fast-moving, and difficult to predict. Prior studies have linked AI anxiety to motivated learning, self-efficacy, career decisions, future career concerns, lifelong learning tendencies, and digital well-being (Wang and Wang, 2022; Chen et al., 2025; Duan et al., 2026; AL-Nasa'h et al., 2026; Cantas et al., 2026; Wang, 2026). This literature treats AI anxiety as a non-clinical response that can emerge when students reassess their knowledge preparation, the future value of work, and career readiness in a changing technological environment.

AI-related psychological pressure can involve several emotions and beliefs. A student may feel anxious about learning AI, changing evaluation standards, moral or social uncertainty, or future employment. Occupational replacement perception is one specific appraisal within this broader response. The STARA awareness framework emphasizes whether individuals believe that smart technology, artificial intelligence, robotics, and algorithms may affect or replace their own work (Brougham and Haar, 2018). It directs attention from objective labor-market forecasts to interpretations of technological change in relation to professional identity and role security (Selenko et al., 2022). Classic discussions of automation have estimated which occupations are vulnerable to computerization (Frey and Osborne, 2017), but these estimates do not fully explain how students in different professional fields experience AI as a psychological and career-related issue.

Existing empirical research indicates that AI-related replacement concerns are more salient in fields where AI can directly intervene in the core tasks through which professional competence is defined. In language-related majors, machine translation, automatic summarization, generative writing, and post-editing technologies can enter translation, interpretation, and foreign-language learning activities. A study of Spanish majors in China showed that when AI was used as a substitute for students' own cognitive work, anxiety and performance-related outcomes differed from cases in which AI functioned as support (Wang et al., 2025). In art design, generative image systems, style transfer, automatic retouching, and layout tools can participate directly in visual ideation and routine production. This has raised concerns about job insecurity, psychological distress, and changes in career exploration among art design professionals and students (Ran and Feng, 2026; Jiang et al., 2026). In computer-related fields, code generation and automated debugging have also led some students to worry about the compression of entry-level programming and web-development jobs (Farooqi et al., 2026). Variation across these fields suggests that AI anxiety and replacement appraisal may depend partly on how directly AI enters a profession's core work.

Vocal music provides a theoretically informative case for examining this domain specificity. AI voice synthesis, virtual singers, intelligent accompaniment, automatic stem separation, audio processing, and AI-assisted vocal evaluation have entered music production, online dissemination, and aspects of music learning (Ouyang, 2025; Bell, 2025; Feng and Tian, 2025; Li et al., 2026). Research with Chinese music students has linked AI readiness with self-efficacy and academic performance, while recent work on generative AI in music education has shown that students' acceptance depends on interactional experience and enjoyment (Wang and Li, 2024; Li, 2026). AI in music education has technical, affective, and motivational dimensions. Teacher scaffolding in Chinese university music classes further shows the importance of situated guidance when new learning demands are introduced (Li and Wang, 2026). Vocal music differs because its professional core extends beyond a reproducible acoustic product. Vocal performance and vocal pedagogy involve embodied vocal production, breath support, register negotiation, resonance control, diction, stylistic interpretation, situated listening, audience interaction, and teachers' bodily and auditory judgment (Li and Ji, 2025; Li et al., 2026). These embodied and relational features distinguish vocal music from fields in which professional output is more easily represented as text, code, or visual objects.

AI already participates in vocal music. It can imitate vocal timbre, assist practice, provide feedback, process sound, and generate materials for music learning. It may also influence adjacent work such as online content production, accompaniment generation, promotional media, or basic audio editing. The four study-specific items address a narrower question: whether vocal music students regard vocal performance as a whole activity that AI could replace or take over. Research on AI art reception suggests that judgments of AI-generated or AI-performed work reflect perceptual quality as well as authorship, authenticity, human effort, intention, and affective connection (Rodero and Lucas, 2023; Shank et al., 2023; Bellaiche et al., 2023; Ansani et al., 2025; van Hees et al., 2025; Samo and Highhouse, 2025). If audiences and practitioners attribute value to human expressive intention and embodied presence, vocal students may experience AI as a source of uncertainty without endorsing whole-activity replacement of human vocal performance.

Employment anxiety provides a useful outcome for examining this issue. The development of an employment anxiety scale for Chinese college graduates indicates that employment anxiety includes both cognitive expectations and emotional or somatic reactions related to job search, occupational fit, and future career uncertainty (Wang and Hu, 2025). For students in arts-related fields, this anxiety may be intensified by competitive career entry, unstable income pathways, and unclear transitions between training and employment (Zhao, 2023). Music students may also experience performance-related anxiety and career expectation pressures (Wang and Yang, 2024), and AI-related employment anxiety has been examined in adjacent student and professional-training populations (Kacay et al., 2026). In the Chinese context, music performance and related arts majors face additional concerns about labor-market demand and professional pathway stability, as reflected in broader employment reports (MyCOS Research Institute, 2024). Vocal students' employment anxiety may arise from general labor-market pressure, artistic career uncertainty, AI-related technological change, and uncertainty about career direction.

Perceived career-direction clarity may be relevant in this setting. Career research has associated clearer expectations about future work with career planning and lower indecision or stress, whereas vague or unstable plans may coincide with stronger uncertainty (Zhang et al., 2022). Vocal students may pursue stage performance, school music teaching, private training, choral direction, cultural institutions, platform-based content production, or digital music work. Students who report less clarity may experience AI-related change as a broad and diffuse concern. A cross-sectional design, however, leaves the direction of this relationship unresolved.

The present study distinguishes broad AI anxiety from a vocal-performance-specific replacement appraisal. The two constructs differ mainly in referent and specificity while retaining conceptual overlap. The administered AI-anxiety items included broad concerns about learning AI, dependence, sociotechnical risks, humanoid AI, and the replacement of humans or people's jobs in general. The separate four-item set asked whether smart technology, AI, robotics, or algorithms could replace or take over vocal performance specifically. A student may endorse general human- or job-replacement concerns without endorsing the view that AI can replace vocal performance. The four-item set therefore operationalized a domain-extension question: whether general replacement concern also applied to a central activity in respondents' own professional field. This possible boundary between a general technological appraisal and a judgment about one's own field requires empirical examination in an embodied arts discipline.

**Figure 1:**
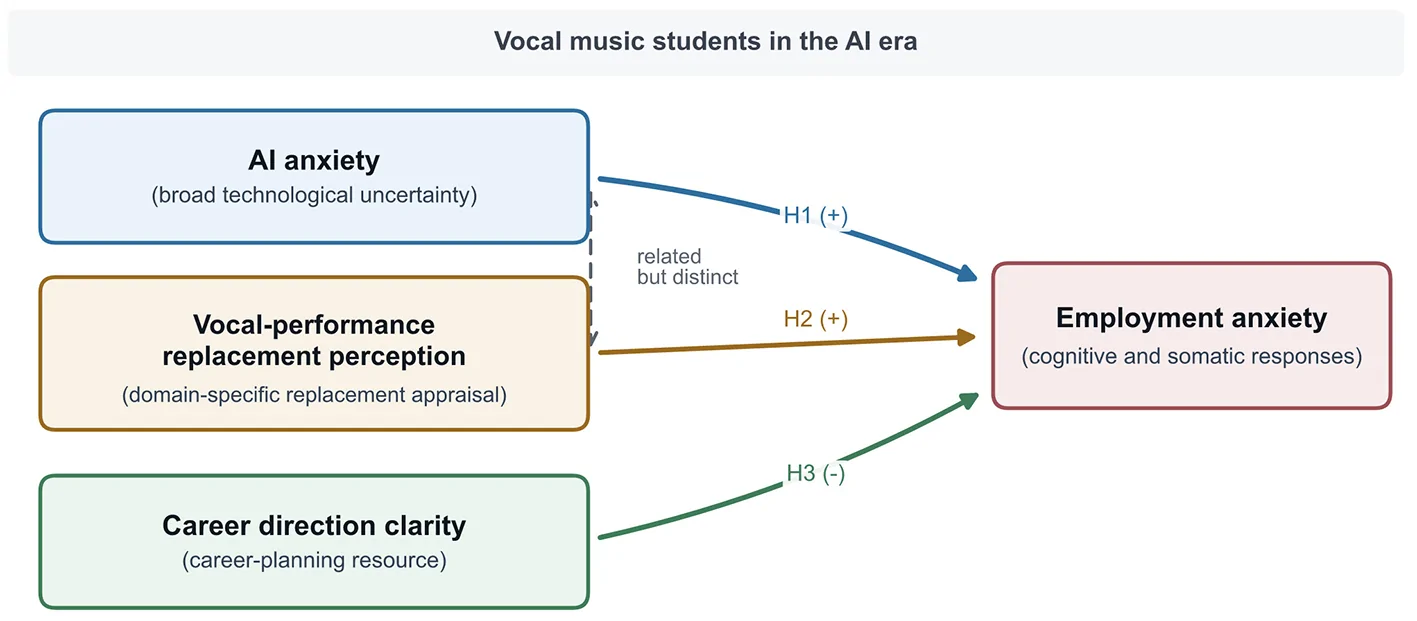
Conceptual model of the core variable relationships. H1–H3 denote hypothesized zero-order associations; RQ1 concerns the additional contribution of vocal-performance replacement perception in the controlled regression model.

Three hypotheses and one research question guided the analyses. H1 proposed that AI anxiety would be positively associated with employment anxiety. H2 proposed that vocal-performance replacement perception would be positively associated with employment anxiety, but less strongly than AI anxiety. H3 proposed that perceived career-direction clarity would be negatively associated with employment anxiety. The study was not preregistered, and the unique contribution of the context-adapted replacement item set was uncertain. RQ1 asked whether vocal-performance replacement perception would explain additional variance in employment anxiety after AI anxiety and the selected grouping variables were considered. An explicitly exploratory profile description examined combinations of relative AI anxiety, vocal-performance replacement perception, and employment anxiety. The conceptual model is shown in Figure 1.

## 2 Materials and methods

### 2.1 Study design, participants, and procedure

This study used a cross-sectional online questionnaire design. The formal survey was administered through Wenjuanxing from June 6 to June 14, 2026. Eligible participants were at least 18 years old and were either current vocal music students or vocal music graduates within three years of graduation. The survey was distributed through professional learning networks, peer referral, and online recruitment channels; some respondents recruited through public online channels received a small RMB 3–5 participation payment. The questionnaire introduction specified the eligibility criteria, explained that participation was voluntary, and stated that no names, student numbers, telephone numbers, precise locations, or other direct identifiers would be solicited. The platform-recorded IP information was used only for duplicate-response and quality checks and was not analyzed as a substantive study variable. The data were used only for group-level statistical analysis.

The formal survey produced 428 submissions. Thirty-one responses that failed the instructed-response attention check were removed. Response completeness and duration were then inspected, and five unusually rapid responses within the single highest-frequency IP cluster were removed in a sensitivity cleaning step. Repeated IP addresses were not used as an automatic exclusion rule because students could share campus, dormitory, studio, or classroom networks. The final analytic sample comprised 392 responses (91.59% of submissions). Sample characteristics are presented in Table 1. The core scale items were mandatory, so there were no missing values for the main variables.

**Table 1:** Sample characteristics.

Variable	Category	*n*	%
Gender	Female	220	56.12
Gender	Male	169	43.11
Gender	Prefer not to say	3	0.77
Education stage/status	Undergraduate, Year 3 or 4	268	68.37
Education stage/status	Bachelor's graduate within 3 years	73	18.62
Education stage/status	Master's student	43	10.97
Education stage/status	Master's graduate within 3 years	8	2.04
School type	Comprehensive university music department	139	35.46
School type	Professional arts/music conservatory	125	31.89
School type	Normal university music department	107	27.30
School type	Overseas higher education institution	9	2.30
School type	Other	12	3.06
AI-use experience	Frequent use	249	63.52
AI-use experience	Occasional use	120	30.61
AI-use experience	Never used	19	4.85
AI-use experience	Systematic learning/training	4	1.03

Before the formal survey, a pilot test yielded 83 valid responses from vocal music students recruited from the researchers' institution and through online channels. The pilot version was used to check item comprehension, questionnaire flow, and internal consistency. Its four replacement items mixed several referents (performance, teaching, content production, employment opportunities, and a 5-to-10-year horizon). Although their internal consistency was acceptable (α = 0.882), participant feedback indicated that the referent was not sufficiently uniform. Based on this feedback, the formal version narrowed all four items to vocal performance and removed the mixed task and time-horizon wording. One doctoral student in psychology and two vocal music teachers then reviewed the revised items for clarity, contextual relevance, and suitability for preliminary survey use; all three considered the revised wording appropriate for the intended inquiry. This was an informal content review rather than a formal content-validity study.

### 2.2 Measures

AI anxiety was assessed with 14 items selected at the questionnaire-design stage from the 20-item Chinese AIAS (Wang et al., 2026), which was adapted from Wang and Wang (2022). The subset was chosen pragmatically to reduce questionnaire burden while retaining content from all four published domains; it was not developed through a formal short-form validation procedure. The administered subset contained four learning-anxiety items, five work-replacement items, three sociotechnical-concern items, and two humanoid-AI/configuration items. One sociotechnical item consolidated closely related examples concerning loss of control, malfunction, and misuse. Responses ranged from 1 (strongly disagree) to 7 (strongly agree), and the mean of the 14 items was used as the broad AI-anxiety composite. Internal consistency was high for the composite (α = 0.937) and acceptable for the four administered domains (learning, α = 0.866; work replacement, α = 0.890; sociotechnical concerns, α = 0.802; humanoid AI/configuration, α = 0.796). Because the full validated 20-item Chinese AIAS was not administered and one item was consolidated, the present measure is described as a study-specific 14-item subset derived from the Chinese AIAS rather than as the complete validated instrument. The administered items are reported in Supplementary material.

Vocal-performance replacement perception was operationalized with a four-item item set informed by the STARA awareness framework (Brougham and Haar, 2018). The original STARA measure asks employees whether smart technologies, AI, robotics, and algorithms may replace their jobs, future organizational roles, or industry employment and uses a five-point response format. During early questionnaire development, Google Gemini (web interface; exact model/version not retained; Google LLC, USA) was used to assist with initial source identification and wording exploration. The resulting Chinese items were reviewed and revised by the authors before pilot testing; they were not a direct translation of the original scale. The items asked whether the same technologies could replace, perform, or take over *vocal performance*; they did not assess vocal teaching, every vocal occupation, or partial task restructuring. Responses ranged from 1 (strongly disagree) to 7 (strongly agree), and item means were averaged. No independent forward translation or back-translation was conducted. The formal item set showed acceptable internal consistency (α = 0.839; corrected item-total correlations = 0.586–0.743). It is therefore treated as a preliminary context-adapted item set, not as the original or a fully validated STARA scale. The Chinese wording and author-produced English renderings are provided in Supplementary Material 1.

Employment anxiety was measured with the 14-item Chinese College Graduates Employment Anxiety Scale (Wang and Hu, 2025). The measure contains nine cognitive-expectation items and five somatic/emotional-response items. Responses ranged from 1 to 5, and higher mean scores indicated stronger employment anxiety. Internal consistency was high for the total score (α = 0.936), the cognitive dimension (α = 0.921), and the somatic/emotional dimension (α = 0.859).

Perceived career-direction clarity was assessed with one item asking respondents how clear they were about their future career direction (1 = very unclear, 5 = very clear). Single-item measures can be useful for narrow, readily understood self-appraisals and can reduce respondent burden (Wanous et al., 1997; Fisher et al., 2016). Nevertheless, this item provides only a limited subjective indicator. It does not measure career decidedness, career adaptability, vocational identity, general decisiveness, or optimism. Demographic and grouping variables included gender, education stage/status, school type, AI-use experience, and intended career direction.

### 2.3 Statistical analysis

Analyses were conducted in Python 3.11 using pandas, SciPy, statsmodels, Pingouin, and semopy. All significance tests were two-sided with α = 0.05. Descriptive statistics, Cronbach's alpha, corrected item-total correlations, and alpha if item deleted were first calculated. To address possible overlap between generalized replacement content within the AIAS subset and the vocal-performance item set, a targeted ordinal confirmatory factor analysis compared a one-factor model with a two-factor model. The analysis used diagonally weighted least squares and included the three AIAS items that explicitly referred to replacing humans or people's jobs together with the four vocal-performance items. HTMT based on the observed item correlations was calculated as supplementary discriminant evidence. These targeted construct-overlap analyses were conducted after the reviewer-raised measurement audit, used the same sample as the main analyses, and were therefore treated as exploratory evidence rather than as independent scale validation.

Pearson correlations were calculated among the central variables. The difference between the two correlations sharing employment anxiety was tested with Williams's test for dependent correlations. Group differences were examined with Welch's ANOVA and Games–Howell *post-hoc* comparisons because category sizes and variances were unequal. These subgroup analyses were exploratory; Games–Howell adjusted the pairwise comparisons within each omnibus test, but no additional familywise correction was applied across the set of grouping variables and outcomes. The hierarchical regression entered collapsed education stage/status, school type, and AI-use experience categories in Block 1 and entered AI anxiety, vocal-performance replacement perception, and career-direction clarity in Block 2. The smallest categories were combined before regression: master's students and recent master's graduates; overseas and other institutions; and frequent users and the four respondents reporting systematic AI training. Continuous-variable standardized coefficients were reported alongside unstandardized coefficients, heteroskedasticity-consistent HC3 standard errors, and 95% confidence intervals.

Model checks included variance inflation factors, residual plots, the Breusch–Pagan test, Durbin–Watson statistic, studentized residuals, leverage, and Cook's distance. Because four observations had externally studentized residuals with absolute values above 3 and residuals were non-normal, the main coefficients were also evaluated with 5,000 percentile-bootstrap resamples (random seed 20260714) and in a sensitivity model excluding those four observations. Two construct-overlap sensitivity models recalculated AI anxiety after (a) excluding the complete five-item work-replacement domain and (b) excluding only the three explicit human/job-replacement items.

The supplementary profile analysis was descriptive and exploratory. Relative AI anxiety was classified using the present sample mean because the 14-item subset has no validated clinical or diagnostic cut-off. High vocal-performance replacement perception was classified above the midpoint of its seven-point response scale. Employment anxiety was classified above education-level reference means reported for the original scale: 2.799 for undergraduate-level respondents and 2.596 for postgraduate-level respondents. The mixed cut-off rationales preclude interpreting the eight combinations as latent classes or diagnostic groups. Because the study used a cross-sectional self-report design, all results are interpreted as associations rather than causal effects.

During manuscript revision, OpenAI's ChatGPT/Codex (GPT-5; OpenAI, USA) assisted with drafting and checking statistical code. The authors executed the scripts locally, checked the variable definitions against the administered questionnaire, and verified the reported values against the saved outputs. The tool did not collect data or make autonomous analytic or interpretive decisions.

### 2.4 Common method bias

All focal variables were collected in one self-report questionnaire, creating a risk of common-method variance. Procedural safeguards included not soliciting direct identifiers, assuring confidentiality and group-level reporting, using neutral instructions that requested responses based on participants' actual experiences, and separating the focal constructs into distinct questionnaire sections (Podsakoff et al., 2003). Harman's single-factor test was applied to the 32 items constituting the three multi-item focal measures and retained only as a coarse diagnostic: the first unrotated factor explained 42.33% of total variance. Because this value is close to or above some commonly used heuristic thresholds and Harman's test cannot establish the absence of method effects, common-method variance cannot be ruled out. The strong association between the broad AI-anxiety composite and employment anxiety is especially susceptible to inflation because both measures contain negatively valenced self-reports. The differentiated results, targeted factor comparison, and overlap sensitivity analyses improve interpretability but do not remove this limitation.

## 3 Results

### 3.1 Descriptive statistics and reliability

Table 2 presents descriptive statistics and reliability coefficients. The 14-item AI-anxiety subset had a mean of 3.460 (SD = 1.299) on a seven-point scale. Vocal-performance replacement perception had a mean of 2.841 (SD = 1.471), below the seven-point scale midpoint. Employment anxiety had a mean of 2.604 (SD = 0.900) on a five-point scale, and perceived career-direction clarity had a mean of 3.457 (SD = 0.967). These values are descriptive rather than diagnostic.

**Table 2:** Descriptive statistics and reliability.

Variable	N	M	SD	Cronbach's α
AI anxiety	392	3.460	1.299	0.937
Vocal-performance replacement perception	392	2.841	1.471	0.839
Employment anxiety	392	2.604	0.900	0.936
Career direction clarity	392	3.457	0.967	-

Item evidence is summarized in Table 3. Corrected item-total correlations ranged from 0.510 to 0.764 for the AI-anxiety subset, from 0.586 to 0.743 for the vocal-performance item set, and from 0.527 to 0.762 for employment anxiety. In the targeted ordinal factor comparison, the two-factor model separating three explicit general-replacement AIAS items from the four vocal-performance items showed better fit than the one-factor model; the unscaled chi-square difference was Δχ^2^(1) = 14.00, *p* < 0.001. With incremental fit indices bounded at 1.00 for reporting, the two-factor model showed CFI = 1.000, TLI = 1.000, RMSEA = 0.000, and a standardized factor correlation of 0.541; the corresponding one-factor model had CFI = 0.953, TLI = 0.929, and RMSEA = 0.037. HTMT based on observed item correlations was 0.517. Because the comparison was targeted, *post hoc*, and conducted in the analytic sample, these results provide only sample-specific evidence of referent separation and do not constitute full validation of either study-specific item set.

**Table 3:** Item evidence and targeted construct comparison.

Measure	CITC range	α if item deleted	Supplementary evidence
14-item AI-anxiety subset	0.510–0.764	0.931–0.938	Four administered domains: α = 0.796–0.890
Vocal-performance replacement item set	0.586–0.743	0.764–0.832	Two-factor vs. one-factor: Δχ^2^(1) = 14.00, *p* < 0.001; HTMT = 0.517
Employment anxiety	0.527–0.762	0.929–0.935	Cognitive α = 0.921; somatic/emotional α = 0.859

### 3.2 Correlations among key variables

**Figure 2:**
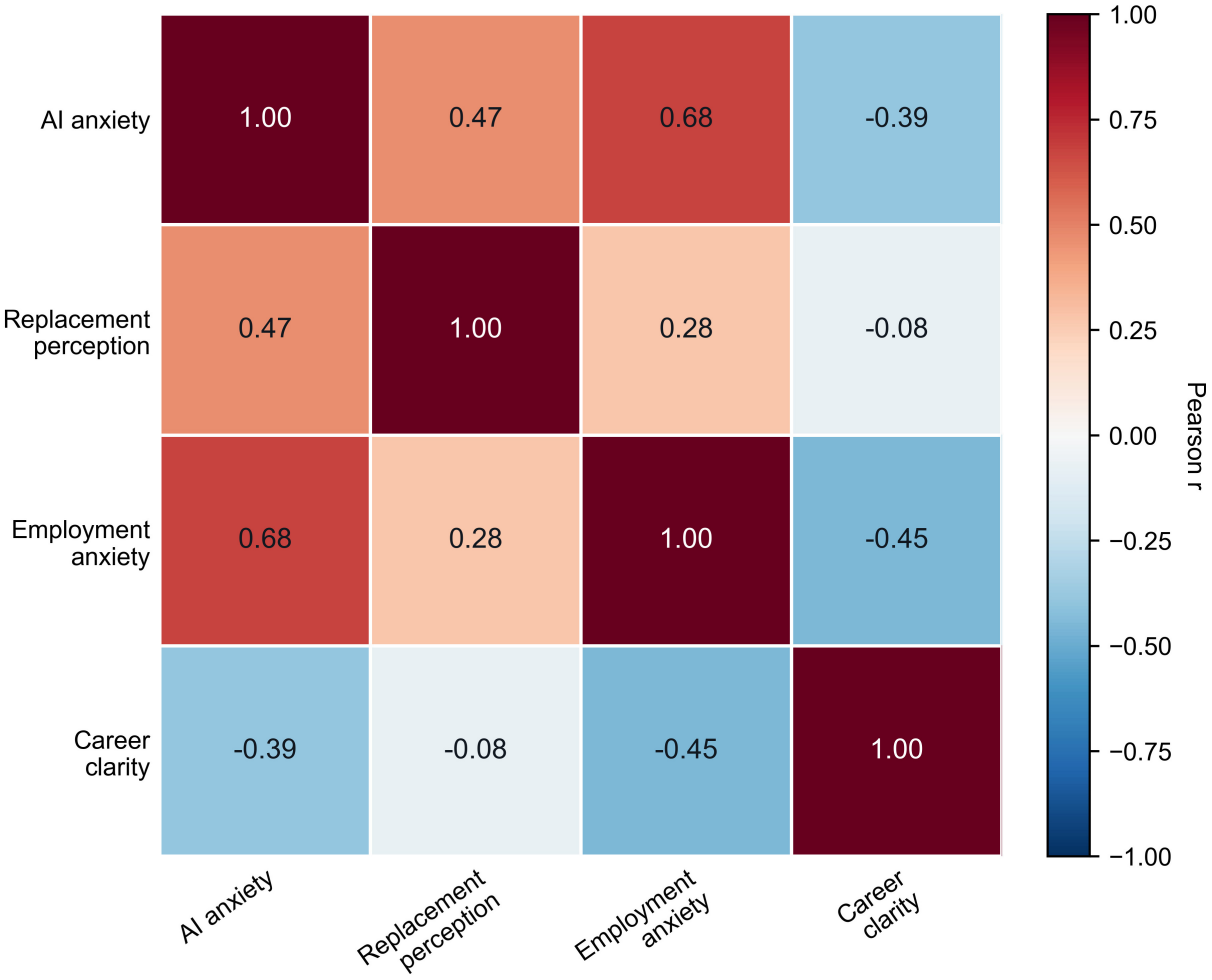
Correlations among key variables. In the figure, replacement perception denotes vocal-performance replacement perception, and career clarity denotes perceived career-direction clarity.

Table 4 and Figure 2 show the zero-order correlations. AI anxiety was positively correlated with employment anxiety (*r* = 0.678, *p* < 0.001), supporting H1. Vocal-performance replacement perception was also positively correlated with employment anxiety (*r* = 0.277, *p* < 0.001). Williams's test confirmed that the AI-anxiety correlation was stronger than the replacement-perception correlation, *t*_(389)_ = 10.23, *p* < 0.001, supporting H2.

**Table 4:** Correlations among the main variables.

Variable 1	Variable 2	r	*p*	*N*
AI anxiety	Vocal-performance replacement perception	0.468	< 0.001	392
AI anxiety	Employment anxiety	0.678	< 0.001	392
AI anxiety	Career direction clarity	–0.386	< 0.001	392
Vocal-performance replacement perception	Employment anxiety	0.277	< 0.001	392
Vocal-performance replacement perception	Career direction clarity	–0.077	0.129	392
Employment anxiety	Career direction clarity	–0.449	< 0.001	392

AI anxiety was positively correlated with vocal-performance replacement perception (*r* = 0.468, *p* < 0.001), indicating overlap without interchangeability. Perceived career-direction clarity was negatively correlated with employment anxiety (*r* = –0.449, *p* < 0.001), supporting H3 at the bivariate level. Career-direction clarity was also negatively correlated with AI anxiety (*r* = –0.386, *p* < 0.001), but not significantly correlated with vocal-performance replacement perception (*r* = –0.077, *p* = 0.129).

### 3.3 Group differences

The robust group comparisons are shown in Table 5. Gender comparisons were limited to the 220 women and 169 men because only three respondents selected “prefer not to say.” No gender difference was significant. AI-use experience also showed no significant differences after the smallest category was combined with frequent use. This null result concerns broad frequency categories only; it does not show that the depth, purpose, competence, or specific tools involved in AI use are irrelevant.

**Table 5:** Group differences in the main variables.

Grouping variable	Dependent variable	Test statistic	df	*p*	Effect size
Gender (female vs. male)	AI anxiety	*t* = 0.578	344.04	0.563	*d* = 0.060
Gender (female vs. male)	Vocal-performance replacement	*t* = −0.392	342.68	0.695	*d* = 0.041
Gender (female vs. male)	Employment anxiety	*t* = 0.965	365.81	0.335	*d* = 0.098
Education stage/status (3 groups)	AI anxiety	*F* = 1.547	2, 117.35	0.217	$\eta_{p}^{2}=0.007$
Education stage/status (3 groups)	Vocal-performance replacement	*F* = 1.711	2, 116.41	0.185	$\eta_{p}^{2}=0.007$
Education stage/status (3 groups)	Employment anxiety	*F* = 7.345	2, 113.44	0.001	$\eta_{p}^{2}=0.036$
School type (4 groups)	AI anxiety	*F* = 1.471	3, 87.29	0.228	$\eta_{p}^{2}=0.008$
School type (4 groups)	Vocal-performance replacement	*F* = 4.717	3, 88.35	0.004	$\eta_{p}^{2}=0.040$
School type (4 groups)	Employment anxiety	*F* = 9.738	3, 86.82	< 0.001	$\eta_{p}^{2}=0.061$
AI-use experience (3 groups)	AI anxiety	*F* = 0.839	2, 52.27	0.438	$\eta_{p}^{2}=0.004$
AI-use experience (3 groups)	Vocal-performance replacement	*F* = 0.177	2, 48.57	0.838	$\eta_{p}^{2}=0.001$
AI-use experience (3 groups)	Employment anxiety	*F* = 2.714	2, 50.14	0.076	$\eta_{p}^{2}=0.013$

Education stage/status was associated with employment anxiety, with a small effect size. Games–Howell comparisons indicated that recent bachelor's graduates reported higher employment anxiety than 3rd- or 4th-year undergraduates (mean difference = 0.425, *p* = 0.001, Hedges' *g* = 0.470). School type was associated with both vocal-performance replacement perception and employment anxiety. Students in professional arts/music conservatories reported higher replacement perception than students in comprehensive-university and normal-university music departments, whereas their employment-anxiety mean was lower than both groups. Full group means, standard deviations, and *post-hoc* comparisons are provided in Supplementary Section S8. These subgroup findings are exploratory; the smallest original categories were collapsed for the inferential and regression analyses, and no institutional mechanism was directly measured.

### 3.4 Dimensions of employment anxiety

The supplementary dimension analysis is presented in Table 6. Cognitive-expectation scores were higher than somatic/emotional-response scores and were somewhat more strongly correlated with AI anxiety. Descriptively, employment anxiety in this sample was more evident in anticipatory concerns about job prospects, ability requirements, and future development than in the measured bodily symptoms. This comparison does not establish a temporal or psychological mechanism.

**Table 6:** Employment anxiety dimensions.

Employment anxiety dimension	M	SD	Cronbach's α	Correlation with AI anxiety
Cognitive expectation	2.785	0.978	0.921	0.674
Somatic/emotional response	2.279	0.929	0.859	0.560

The dimension-level pattern is consistent with a future-oriented appraisal interpretation: students may be considering whether their training remains adequate, whether new competencies will be required, and whether AI will change expectations within education and employment. Because these appraisals were not directly measured, this remains a theoretical interpretation of the observed score pattern.

### 3.5 Regression analysis

The hierarchical regression results are shown in Tables 7, 8. Education stage/status, school type, and AI-use experience explained 9.4% of the variance in Block 1. Adding AI anxiety, vocal-performance replacement perception, and career-direction clarity increased explained variance by 44.6 percentage points, Δ*F*_(3, 381)_ = 123.14, *p* < 0.001. The full model explained 54.0% of the variance (*R*^2^ = 0.540, adjusted *R*^2^ = 0.528).

**Table 7:** Regression model summary.

Model	*N*	*R* ^2^	Adjusted *R*^2^	*ΔR* ^2^	*F*	*ΔF*	*p*
Block 1: grouping variables	392	0.094	0.077	–	5.683	–	< 0.001
Block 2: central variables	392	0.540	0.528	0.446	44.716	123.137	< 0.001

**Table 8:** Regression coefficients for employment anxiety.

Explanatory variable	B	HC3 SE	Standardized β	*t*	*p*	95% CI
Recent bachelor's graduate (vs. undergraduate)	0.243	0.090	–	2.695	0.007	[0.066, 0.420]
Postgraduate student/recent graduate (vs. undergraduate)	0.020	0.102	–	0.196	0.844	[–0.181, 0.221]
Normal-university music department (vs. comprehensive)	0.050	0.089	–	0.560	0.576	[–0.125, 0.224]
Other/overseas institution (vs. comprehensive)	-0.225	0.183	–	-1.230	0.220	[–0.584, 0.134]
Professional arts/music conservatory (vs. comprehensive)	-0.298	0.075	–	–3.979	< 0.001	[–0.445, –0.151]
Frequent/systematic AI exposure (vs. occasional)	-0.003	0.074	–	–0.044	0.965	[–0.149, 0.142]
Never used AI (vs. occasional)	0.066	0.181	–	0.362	0.717	[–0.290, 0.421]
AI anxiety	0.408	0.035	0.589	11.745	< 0.001	[0.340, 0.477]
Vocal-performance replacement perception	0.006	0.028	0.010	0.223	0.824	[–0.048, 0.061]
Perceived career-direction clarity	-0.162	0.045	–0.174	–3.642	< 0.001	[–0.250, –0.075]

In the full model, AI anxiety had a positive coefficient (β = 0.589, HC3 95% CI for *B* [0.340, 0.477], *p* < 0.001), and perceived career-direction clarity had a negative coefficient (β = -0.174, 95% CI for *B* [–0.250, –0.075], *p* < 0.001). Vocal-performance replacement perception did not provide additional independent explanatory information (β = 0.010, 95% CI for *B* [–0.048, 0.061], *p* = 0.824), answering RQ1. Its significant zero-order association remained relevant, but it was not independently detectable after the correlated broad AI-anxiety composite and controls were included.

Diagnostics supported the stability of the coefficient pattern. All VIFs were below 1.55, the Breusch–Pagan test for the full model was non-significant (*p* = 0.712), and the Durbin–Watson statistic was 1.79. Four observations had absolute externally studentized residuals above 3, and residual normality was imperfect. Given this residual behavior, HC3 standard errors were retained. Bootstrap percentile intervals based on 5,000 resamples were [0.338, 0.471] for AI anxiety, [–0.049, 0.059] for vocal-performance replacement perception, and [–0.253, –0.079] for career-direction clarity. Excluding the four observations did not change the pattern. The construct-overlap sensitivity analyses are presented in Table 9; both alternative AI-anxiety specifications yielded the same coefficient pattern.

**Table 9:** Construct-overlap sensitivity analyses.

AI-anxiety specification	*R* ^2^	AI-anxiety β	*p*	Vocal replacement β	*p*	Clarity β	*p*
14-item administered subset	0.540	0.589	< 0.001	0.010	0.824	–0.174	< 0.001
Exclude complete work-replacement domain	0.511	0.545	< 0.001	0.038	0.416	–0.193	< 0.001
Exclude three explicit human/job-replacement items	0.536	0.580	< 0.001	0.018	0.683	–0.182	< 0.001

### 3.6 Supplementary profile analysis

**Figure 3:**
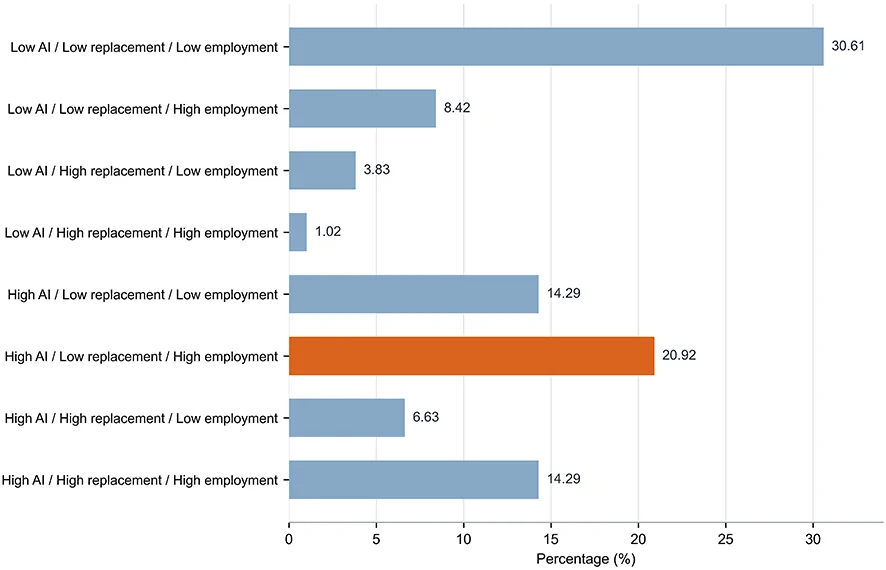
Exploratory descriptive profile distribution. In the figure, AI refers to relative AI anxiety based on the sample-mean threshold, replacement refers to vocal-performance replacement perception based on the response-scale midpoint, and employment refers to employment anxiety based on education-level reference means.

The exploratory profile description is shown in Table 10 and Figure 3. “Relatively high” AI anxiety means above the present sample mean and is not an absolute or clinical classification. Students above this cut-off accounted for more than half of the sample, whereas approximately one quarter scored above the midpoint for vocal-performance replacement perception. The combination of relatively high AI anxiety, replacement perception at or below the midpoint, and employment anxiety above the education-level reference mean accounted for 20.92% of the sample.

**Table 10:** Exploratory distribution across AI anxiety, replacement perception, and employment anxiety.

Relatively high AI anxiety	High replacement perception	High employment anxiety	*n*	%
No	No	No	120	30.61
No	No	Yes	33	8.42
No	Yes	No	15	3.83
No	Yes	Yes	4	1.02
Yes	No	No	56	14.29
Yes	No	Yes	82	20.92
Yes	Yes	No	26	6.63
Yes	Yes	Yes	56	14.29

The group below all three cut-offs accounted for 30.61% of the sample, showing that the sample was not uniformly above the selected reference points. Possible explanations include differences in career stage, perceived career options, learning environments, and social support, none of which can be inferred from this descriptive classification. Because the three cut-offs have different rationales, these combinations are not latent profiles, diagnoses, or tests of the study hypotheses.

## 4 Discussion

### 4.1 Principal findings

This study examined how two AI-related appraisals were associated with employment anxiety among vocal music students and recent graduates. AI anxiety had a strong zero-order association with employment anxiety and retained a positive coefficient after education stage/status, school type, AI-use experience, vocal-performance replacement perception, and career-direction clarity were considered. Vocal-performance replacement perception had a weaker zero-order association and did not explain additional variance in the full model. The difference between the zero-order correlations was statistically significant. The same pattern remained when the AI-anxiety composite excluded either its complete work-replacement domain or only its explicit human/job-replacement items. Perceived career-direction clarity was negatively associated with employment anxiety, although it was measured with one item.

The findings complicate the assumption that AI-related employment pressure in every discipline reflects a direct replacement belief. The present operationalization focused narrowly on whether AI could replace or take over vocal performance as a whole; it did not cover every task or occupation available to vocal graduates. Within that boundary, the association pattern was more closely tied to broad AI anxiety than to explicit vocal-performance replacement perception. The cross-sectional design leaves temporal ordering unresolved, and the general employment difficulty facing vocal graduates remains an unmeasured alternative source of employment anxiety.

### 4.2 AI anxiety and employment anxiety

The strong association between AI anxiety and employment anxiety is consistent with research connecting AI anxiety with career-related cognition and learning behavior (Chen et al., 2025; Duan et al., 2026; Wang, 2026). The administered AI-anxiety subset included learning difficulty, work-replacement concerns, sociotechnical risks, and fear of humanoid AI. It may capture a broad negative appraisal of rapid technological change. Students who perceive AI as difficult to learn, difficult to keep up with, or socially disruptive may also report stronger employment concerns. However, both variables were negatively valenced self-reports collected at one time point. Common-method variance, trait anxiety, neuroticism, or generalized negative affect could inflate their correlation; none was measured here.

For vocal music students, plausible sources of uncertainty include changing training practices, audience expectations, digital-platform work, and future competence requirements. The higher cognitive-expectation score relative to the somatic/emotional score is consistent with future-oriented concern, but the survey did not directly measure these proposed sources. Dedicated items, interviews, or longitudinal designs are needed to test them as possible mechanisms.

### 4.3 Broad AI anxiety and domain-specific replacement perception

The weaker conditional coefficient for vocal-performance replacement perception is central to the study's interpretation. Its zero-order correlation with employment anxiety was significant, and the two AI-related measures were moderately correlated. In the regression, however, this four-item appraisal did not add independently detectable information once the broader AI-anxiety composite and controls were included. The same pattern remained after overlapping work-replacement content was removed from the AI-anxiety score. This comparison addresses a domain-extension question rather than measuring replacement belief twice: endorsement of general human- or job-replacement concerns need not entail endorsement of AI replacing vocal performance. The present data show that these appraisals were related but not interchangeable; they do not establish that vocal music is an exception across occupations or disciplines.

The result is compatible with a domain-specific reading of STARA awareness. In translation, design, or coding, AI systems can directly enter core symbolic production tasks. Vocal performance also involves technological mediation, but students may evaluate replacement in relation to bodily production, expressive intention, and situated performance. Because the design did not compare disciplines, the evidence is limited to a within-sample distinction between generalized AI concern and vocal-performance replacement appraisal.

### 4.4 Interpreting vocal-performance replacement appraisal

The mean for vocal-performance replacement perception was below the response-scale midpoint. This result concerns the replacement of vocal performance as a whole, the referent used in all four items. Descriptively, 138 of the 220 students classified as relatively high in AI anxiety (62.73%) nevertheless scored at or below the midpoint on vocal-performance replacement perception. Using the exploratory profile cutoffs, 82 of the 138 students above both the AI-anxiety and employment-anxiety cutoffs (59.42%) also remained at or below the replacement-perception midpoint. Generalized concern about AI, even when accompanied by elevated employment anxiety under the study's descriptive criterion, did not necessarily extend to a strong endorsement of replacement within students' own performance domain.

Research on AI art reception offers one possible explanation. Listeners and viewers often evaluate AI-generated or AI-performed art differently when they attribute different authorship, effort, intention, or authenticity to it (Rodero and Lucas, 2023; Shank et al., 2023; Bellaiche et al., 2023; Ansani et al., 2025; van Hees et al., 2025; Samo and Highhouse, 2025). Vocal performance is also an embodied and social encounter in which audiences can respond to effort, vulnerability, emotion, and presence. Students may believe that human performers retain value even when synthetic voices become perceptually convincing. The survey did not measure embodiment, authenticity, audience preference, or human-presence beliefs, so this account remains theoretical.

Related arguments may apply to vocal pedagogy, where listening, bodily demonstration, individualized imagery, and relational judgment are central. However, the four items in this study referred only to vocal performance. No inference about perceived replacement of vocal teachers can be drawn from these scores, and future research should assess performance, teaching, audio production, and other occupational tasks separately.

### 4.5 Perceived career-direction clarity as a negative correlate

Perceived career-direction clarity was negatively associated with employment anxiety after the grouping variables and both AI-related measures were considered. This is consistent with research linking future work self and career planning with lower indecision and stress (Zhang et al., 2022). A clear direction may help students translate diffuse uncertainty into concrete preparation. The reverse ordering is also possible: students with stronger employment anxiety may experience their plans as less clear.

For vocal music students, perceived career direction can refer to performance, school teaching, private instruction, choral direction, cultural programming, or platform-based content work, each with a different relationship to AI. However, the single item used here does not capture career decidedness, adaptability, vocational identity, or the quality of a plan. Its coefficient could partly reflect general decisiveness, optimism, or negative affect. The finding supports further multidimensional measurement of career development; it does not establish a protective effect of clarity.

### 4.6 Group differences and educational contexts

The exploratory group comparisons suggest that education stage/status and school type deserve further study. Recent bachelor's graduates reported more employment anxiety than upper-year undergraduates. This pattern is consistent with greater proximity to labor-market transition, but cohort composition and other unmeasured differences may also contribute. In the controlled model, the recent-graduate coefficient remained positive.

Students in professional arts/music conservatories reported higher vocal-performance replacement perception but lower employment anxiety than students in comprehensive- and normal-university music departments. This combination cautions against treating replacement appraisal and employment anxiety as interchangeable. Institutional differences in curriculum, career routes, selection, or professional identity are plausible explanations, but none was measured. The original overseas (*n* = 9) and other (*n* = 12) categories were too small for stable separate inference and were combined in the robust and regression analyses.

### 4.7 Educational implications

The findings support several cautious implications for vocal education. First, AI literacy can be integrated into vocal training beyond tool operation, including the limits of synthetic voice systems, copyright and voice-right issues, algorithmic evaluation, and human–AI collaboration. The present data do not show that such instruction reduces anxiety, but they identify a need for interventions that can be evaluated prospectively.

Second, teaching can help students distinguish generalized technological concern from specific evidence about performance tasks. Discussion of embodied production, expressive intention, audience relations, and the actual capabilities of current tools may enable more differentiated professional judgments without presuming that human performance is permanently immune to technological change.

Third, career guidance may need to address ordinary labor-market difficulty and AI-related uncertainty together. Stage-sensitive support could include career mapping, portfolio development, teaching credentials, and informed use of AI-assisted practice or production tools. The school-type differences further suggest that institutional context should be examined before assuming one intervention will fit all vocal students.

### 4.8 Theoretical contribution

The study provides preliminary evidence for treating a broad AI-anxiety composite and a vocal-performance-specific replacement appraisal as related but distinct measures. Their moderate correlation, targeted two-factor solution, and different regression behavior support examining both generalized and domain-referenced appraisals. More specifically, the design makes visible a domain boundary that a general replacement item cannot resolve: a respondent can express concern about AI replacing humans or jobs while withholding the same judgment about a central activity in their own professional field. Both measures were study-specific subsets or adaptations, and no cross-disciplinary comparison was made; the contribution is therefore a measurement and research-design proposition for further testing rather than evidence that vocal music is an exception across occupations or disciplines.

The study also connects music-education technology research with career psychology. Recent studies of music students have emphasized AI readiness, adoption, enjoyment, and teacher scaffolding (Wang and Li, 2024; Li, 2026; Li and Wang, 2026). The present results add a complementary question: how generalized AI concern, domain-specific replacement appraisal, and employment anxiety coexist during training and early career transition.

## 5 Limitations and future research

Several limitations should be noted. First, convenience, peer-referral, and online recruitment do not provide a probability sample. Although eligibility instructions, an attention check, response-duration screening, and repeated-IP sensitivity cleaning were used, the professional identity of every respondent could not be independently verified. The sample is not representative of all vocal music students in China, and the findings may not generalize to other cultural or labor-market contexts.

Second, all central measures were collected simultaneously by self-report. The design cannot determine whether AI anxiety precedes employment anxiety, whether employment anxiety heightens AI concern, or whether both reflect generalized negative affect. No marker variable, trait-anxiety measure, general anxiety measure, or neuroticism measure was included. Harman's first factor accounted for 42.33% of variance. This value warrants caution, but Harman's test is a coarse diagnostic rather than an estimate of the amount of bias in any particular coefficient; it therefore cannot establish either the absence or the magnitude of common-method effects. The strong AI-anxiety–employment-anxiety correlation is the result most plausibly susceptible to inflation because both constructs were assessed at the same time using negatively valenced self-report items. Its observed magnitude may consequently be overestimated, although the present data cannot quantify the degree of inflation. Future studies should combine procedural and statistical remedies, including temporal or psychological separation of predictor and outcome measurement, inclusion of a theoretically unrelated marker variable, counterbalancing of item order where appropriate, validated measures of trait anxiety or negative affect, and multi-source or behavioral indicators. Longitudinal designs would further help distinguish shared response tendencies from temporal relations among the constructs. The anonymity, confidentiality assurances, neutral instructions, and separation of focal constructs into questionnaire sections used here may have reduced evaluation apprehension, but they cannot eliminate common-method variance.

Third, neither AI-related measure was administered in its complete published form. The AI-anxiety score was based on 14 selected items from the 20-item Chinese AIAS and included one consolidated sociotechnical item. The four vocal-performance items were informed by STARA but used a different referent, wording, and seven-point response format; their initial source identification and wording exploration included generative-AI assistance, and they were not created through independent forward translation and back-translation. Their internal consistency, targeted two-factor result, and HTMT provide preliminary study-specific evidence only. The item set also assessed perceived replacement of vocal performance as a whole rather than partial tasks, teaching, or all occupations open to vocal graduates. Independent development work should use systematic item generation, bilingual review where relevant, formal content-validity indices, external validation samples, test–retest reliability, and measurement-invariance tests.

Fourth, perceived career-direction clarity was measured with one item and may partly reflect decisiveness, optimism, or negative affect rather than career-specific clarity. The study also did not measure AI literacy, actual competence, resilience, socioeconomic background, family or social support, labor-market exposure, or objective employment outcomes. These omitted variables limit interpretation of both the regression coefficients and the exploratory group differences.

Fifth, the study was not preregistered. The hypotheses, RQ1, construct-overlap checks, robust subgroup analyses, and profile description are transparent analyses of the present dataset, not preregistered confirmatory tests. The profile cut-offs used different rationales and were retained only for description; they do not identify latent classes or clinically high-anxiety groups.

## 6 Conclusion

Among the AI-related variables examined, employment anxiety was more strongly associated with the 14-item broad AI-anxiety subset than with the four-item vocal-performance replacement appraisal. Vocal-performance replacement perception had a positive zero-order correlation with employment anxiety but did not add independently detectable information after broad AI anxiety, grouping variables, and perceived career-direction clarity were considered. This pattern remained after removing overlapping replacement content from the AI-anxiety score. Perceived career-direction clarity was a negative correlate, but its single-item measurement limits interpretation.

The findings support a domain-specific and measurement-conscious approach to AI-related student psychology. General concern about AI did not necessarily translate into an equally strong belief that AI would replace vocal performance. At the same time, the study does not establish causal direction, validate a new scale, or show that AI replacement concerns are irrelevant. Replication with complete validated measures, stronger sampling, general-anxiety controls, and longitudinal or comparative designs is required.

## Data Availability

The datasets presented in this article are not readily available because the raw dataset contains questionnaire responses from human participants and includes potentially sensitive contextual information, including demographic, educational, career-related, and AI-related psychological responses. The dataset is not publicly available and is not shared by default because unrestricted sharing could increase privacy risks and go beyond the scope of the consent provided by participants. Aggregated results and measurement information are provided in the manuscript and Supplementary material. Requests to access the datasets should be directed to Kehang Li, the designated data custodian, at likehang1209@gmail.com.
