# Supplementary material for: Is employment anxiety among vocal music students associated with AI replacement concerns? The roles of AI anxiety and vocal-performance replacement perception
Source: Front Psychol. 2026 Aug 14;17:1923159. doi: 10.3389/fpsyg.2026.1923159 (PMC13522129; doi:10.3389/fpsyg.2026.1923159)
Supplement: Supplementary file 1 [file Data_Sheet_1.pdf]

Supplementary Material 1

Measures and Vocal-Context Adapted Items

Manuscript title: Is employment anxiety among vocal music students associated with AI replacement concerns? The roles of AI anxiety and vocal-performance replacement perception

Purpose. This file provides measurement information for transparency and reproducibility. Because the Artificial Intelligence Anxiety Scale and the Chinese College Graduates Employment Anxiety Scale are published instruments, their full item text is not reproduced. The vocal-context adapted replacement items and the single-item career-direction clarity measure are reproduced because they were adapted or specified for the present study.

Translation note. The formal questionnaire was administered in Chinese. The English wording below is an author-produced rendering of the administered Chinese items and should not be interpreted as an independently validated English-language scale.

Table S1. Overview of measures

Construct	Source or basis	Items disclosed here	Response format	Scoring direction
AI anxiety	Study-specific 14-item subset derived from the Chinese AIAS (Wang et al., 2026), based on Wang and Wang (2022).	Published item text not reproduced; source, format, and scoring are reported.	7-point Likert-type scale	Higher scores indicate stronger AI anxiety.
Vocal-performance replacement perception	Four vocal-context items informed by the STARA awareness framework (Brougham and Haar, 2018).	All four context-adapted items are reproduced in Table S2.	7-point Likert-type scale	Higher scores indicate stronger perceived replaceability of vocal performance.
Employment anxiety	Chinese College Graduates Employment Anxiety Scale (Wang and Hu, 2025).	Published item text not reproduced; source, format, and scoring are reported.	5-point Likert-type scale	Higher scores indicate stronger employment anxiety.
Career-direction clarity	Single-item self-appraisal used in the present study.	The item is reproduced in Table S3.	5-point Likert-type scale	Higher scores indicate clearer perceived career direction.

Table S2. Vocal-context adapted replacement-perception items

Instructions and response format. Respondents rated each item on a 7-point Likert-type scale from 1 (strongly disagree) to 7 (strongly agree). The items were reviewed for clarity and contextual relevance by one doctoral student in psychology and two vocal music teachers before formal use.

Item	Administered Chinese item	Author-produced English rendering
ORP1	我担心声乐表演将被智能技术、人工智能、机器人或算法取代。	I worry that vocal performance will be replaced by smart technology, artificial intelligence, robotics, or algorithms.
ORP2	我觉得声乐表演可以由智能技术、人工智能、机器人或算法来完成。	I think that vocal performance can be performed by smart technology, artificial intelligence, robotics, or algorithms.
ORP3	我认为智能技术、人工智能、机器人或算法将接管声乐表演。	I believe that smart technology, artificial intelligence, robotics, or algorithms will take over vocal performance.
ORP4	我相信未来声乐表演将被智能技术、人工智能、机器人或算法所取代。	I believe that vocal performance will be replaced by smart technology, artificial intelligence, robotics, or algorithms in the future.

Table S3. Single-item career-direction clarity measure

Measure	Administered Chinese item	Author-produced English rendering
Career-direction clarity	你对自己未来职业方向的清晰程度：	How clear are you about your future career direction?

Reliability and preliminary item evidence

AI anxiety. The 14-item AI-anxiety subset showed high internal consistency in the present sample (Cronbach's alpha = 0.937).

Vocal-performance replacement perception. The four context-adapted items showed acceptable internal consistency (Cronbach's alpha = 0.839). Corrected item-total correlations ranged from 0.586 to 0.743. Exploratory one-factor evidence indicated that the four items explained 67.74% of the variance, with factor loadings ranging from 0.757 to 0.871. These results constitute study-specific preliminary evidence and do not establish validation of a new scale or equivalence with the original STARA measure.

Employment anxiety. The employment-anxiety measure showed high internal consistency in the present sample (Cronbach's alpha = 0.936).

References

Brougham, D., and Haar, J. (2018). Smart Technology, Artificial Intelligence, Robotics, and Algorithms (STARA): Employees' perceptions of our future workplace. Journal of Management & Organization, 24, 239 – 257. doi: 10.1017/jmo.2016.55

Wang, D., Shang, L., Sun, L., Liu, H., and Wang, K. (2026). Chinesization of Artificial Intelligence Anxiety Scale and its reliability and validity test. Chinese Nursing Research, 40, 1730 – 1735. doi: 10.12102/j.issn.1009-6493.2026.10.017

Wang, W., and Hu, Y. (2025). The development of an employment anxiety scale for Chinese college graduates. Health & Social Care in the Community, 2025, 9964482. doi: 10.1155/hsc/9964482

Wang, Y. Y., and Wang, Y. S. (2022). Development and validation of an artificial intelligence anxiety scale: An initial application in predicting motivated learning behavior. Interactive Learning Environments, 30, 619 – 634. doi: 10.1080/10494820.2019.1674887
